# 15 Years of Success and Still Improving: *Polymers*

**DOI:** 10.3390/polym15040945

**Published:** 2023-02-14

**Authors:** Alexander Böker

**Affiliations:** Lehrstuhl für Polymermaterialien und Polymertechnologie, University of Potsdam, 14476 Potsdam-Golm, Germany; alexander.boeker@iap.fraunhofer.de

In 2009, the inaugural issue of *Polymers* was released. *Polymers* was published as a quarterly journal. The year 2023 marks the 15th anniversary (15th issue) of the journal, and over the years, *Polymers* has published more than 18,500 papers. Today, the published papers receive more than 1,000,000 views per month, with readers in more than 150 countries and geographical regions [1]. We would like to express our gratitude to everyone who has supported *Polymers* over the past several years. We value the contributions of both the authors and the reviewers. The goal of *Polymers* is to help scientists in the field of polymer research to achieve their publication objectives and increase awareness of the latest developments in polymer science, rendering this research openly accessible for the whole scientific community.

Open access (OA) publishing unlocks research that would otherwise be hidden behind paywalls, enabling researchers to share scientific information and data at moderate author charges and ensuring free access for every reader. This creates more equal opportunities among scientific communities around the world [2].

The OA model is not perfect, and there is still room for improvement. Of course, OA is also a business model. However, I encourage readers to ask themselves, honestly, which is preferable? A few million authors (scientists) paying for the publications, or billions of readers paying for access to them? Are extra charges for color images fair, when papers are mostly read online? I am not criticizing other publishing models, but rather, would like to encourage a fair discussion on the advantages and disadvantages. As in many fields, there is never one best model. This discussion is more about the best working and—as far as possible—fairest model. MDPI is a responsible and innovative publisher that acts to benefit scientific communities as a service provider, rather than a product provider. We encourage our readers to spread the word in the scientific community. Instead of talking about us, talk with us! Additionally, and more importantly, try publishing with *Polymers* and consider your own experience.

*Polymers* offers traditional peer review combined with a fast and cost-effective publication process. All *Polymers* articles are peer-reviewed and accepted by our Academic Editors, who are either Editorial Board members or Guest Editors. The names of the editors who accept the articles after peer review are mentioned in each manuscript, and this certainly helps to ensure accountability in the editorial process. Many of our authors opt for open peer review, which renders the review reports openly available alongside the published article [3].

We strive to render the peer review process more rigorous, and so we are always adding new features to help our reviewers. We provide a guideline for the peer review report, and all peer review reports received are checked to ensure that they contain valuable comments for the authors. MDPI takes peer review seriously and holds a yearly Peer Review Week, as well as different webinars, on the subject [4,5,6,7,8]. It is also important that we remember and appreciate the contributions of reviewers who offer the time to help to improve their fields of research. Fair and critical feedback on a submission is as important as a well-written paper on an intriguing topic. In addition, *Polymers* has staff spread across seven offices globally, offering an around-the-clock service to authors and editors in different time zones. We handle administrative tasks (reformatting references, making layout corrections, etc.) to reduce the time spent by authors on issues that can easily be handled by publishers.

*Polymers* is an environment of continuous change, learning, and improvement. Every year, we collect feedback from our Editorial Board members and collaborators, and on this basis, we decide what changes must be made in order to render the publishing process more transparent and to fulfill as many of the needs of the scientific community as possible. In addition, constructive criticism is always welcome, and the *Polymers* team aims to handle all feedback with high priority.

Last year was fruitful but also posed challenges for our journal. Most importantly, the second *Polymers* conference, “New Trends in Polymer Science: Health of the Planet, Health of the People”, was held in Turin, Italy [9]. It was a successful conference, and we were pleased to reconvene with many of our collaborators in person. Such cases remind us of how much we missed these “real-life” conferences during the past few years.

We seized each challenge to better understand how we might provide support for the polymer research community. In 2022, we welcomed 13 new female advisory board members. All are well-respected professors and distinguished scientists, who will further help *Polymers* to improve and ensure the quality of its published works. Moreover, they are role models for a successful career in science. With respect to successful careers, *Polymers* will continue its efforts to support young scientists in particular. We seek to do this through travel grants for conferences, PhD thesis awards, and the bi-annual Young Investigators Award, in addition to the Best Paper and Outstanding Reviewer awards.

We look forward to shaping the next fifteen years of the journal and count on you—our authors, readers, and reviewers—to aid us in this project.
